# Book Review: Telepractice in Audiology

**DOI:** 10.5195/ijt.2016.6198

**Published:** 2016-07-01

**Authors:** BARBARA A. VENTO

**Affiliations:** DEPARTMENT OF COMMUNICATION SCIENCE AND DISORDERS, UNIVERSITY OF PITTSBURGH, PITTSBURGH, PENNSYLVANIA, USA

**Keywords:** Audiology, Cochlear implants, Hearing aid fitting, Tele-audiology, Telepractice, Telerehabilitation

## Abstract

This article presents a review of the book *Telepractice in Audiology*, authored by Emma Rushbrooke MPhil(AUD), BA, DipAud., MAudSA., LSLS. Cert. AVT, RNC, and K. Todd Houston, PhD, CCC-SLP, LSLS Cert. AVT, and 13 contributing authors. This is the first book entirely devoted to tele-audiology. It provides practical information for working with clients across the lifespan and for multiple practice settings. Reviewer Dr. Barbara Vento endorses this work as a comprehensive resource on the topic of teleaudiology for both students and aspiring teleaudiologists.

Emma Rushbrooke and K. Todd Houston and contributing chapter authors have produced an excellent book, *Telepractice in Audiology*. It includes practical information on how to develop and implement a successful telepractice program. The book’s organization is easy to follow with key points and a summary section for individual chapters. The authors address technological requirements, service delivery models, regulatory issues and policy, and future directions.

Chapters 1–3 focus on the history, models of service delivery and how to evaluate various teleaudiology models.

Chapters 4–8 present telepractice models for audiology diagnostics and rehabilitation. These chapters include newborn hearing screenings, general diagnostic procedures, remote programming of cochlear implants, remote hearing aid fitting and follow up care, and telerehabilitation.

Chapters 9–11 propose future directions for telepractice and underscores the need for continuing research to support expanded teleaudiology services to patients and clients.

In conclusion*, Telepractice in Audiology* is an excellent textbook for AuD students. It is a credible and clear source of information for audiologists who might consider incorporating tele-audiology in their practice settings, as well as for experienced tele-audiologists who wish to gain additional perspectives on the range of current practice. The contents of this book will also be of interest to speech-language pathologists, policy makers, and payers. Rushbrooke and Houston have produced a book that is comprehensive, evidence-based, well written, and strong on references.
